# Effects of first-line diabetes therapy with biguanides, sulphonylurea and thiazolidinediones on the differentiation, proliferation and apoptosis of islet cell populations

**DOI:** 10.1007/s40618-021-01620-6

**Published:** 2021-06-30

**Authors:** D. Sarnobat, R. C. Moffett, P. R. Flatt, A. I. Tarasov

**Affiliations:** School of Biomedical Sciences, Ulster University, Cromore Road, Coleraine, BT52 1SA Northern Ireland UK

**Keywords:** Oral hypoglycaemic agents, Streptozotocin-induced diabetes, α-cells, Beta-cell proliferation, Transdifferentiation

## Abstract

**Aims:**

Metformin, rosiglitazone and sulfonylureas enhance either insulin action or secretion and thus have been used extensively as early stage anti-diabetic medication, independently of the aetiology of the disease. When administered to newly diagnosed diabetes patients, these drugs produce variable results. Here, we examined the effects of the three early stage oral hypoglycaemic agents in mice with diabetes induced by multiple low doses of streptozotocin, focusing specifically on the developmental biology of pancreatic islets.

**Methods:**

Streptozotocin-treated diabetic mice expressing a fluorescent reporter specifically in pancreatic islet α-cells were administered the biguanide metformin (100 mg/kg), thiazolidinedione rosiglitazone (10 mg/kg), or sulfonylurea tolbutamide (20 mg/kg) for 10 days. We assessed the impact of the treatment on metabolic status of the animals as well as on the morphology, proliferative potential and transdifferentiation of pancreatic islet cells, using immunofluorescence.

**Results:**

The effect of the therapy on the islet cells varied depending on the drug and included enhanced pancreatic islet β-cell proliferation, in case of metformin and rosiglitazone; de-differentiation of α-cells and β-cell apoptosis with tolbutamide; increased relative number of β-cells and bi-hormonal insulin + glucagon + cells with metformin. These effects were accompanied by normalisation of food and fluid intake with only minor effects on glycaemia at the low doses of the agents employed.

**Conclusions:**

Our data suggest that metformin and rosiglitazone attenuate the depletion of the β-cell pool in the streptozotocin-induced diabetes, whereas tolbutamide exacerbates the β-cell apoptosis, but is likely to protect β-cells from chronic hyperglycaemia by directly elevating insulin secretion.

**Supplementary Information:**

The online version contains supplementary material available at 10.1007/s40618-021-01620-6.

## Introduction

Type 2 diabetes (T2D) is a metabolic disease of increasing incidence fuelled by obesity and ageing demographics [1]. Corresponding to > 80% of the less common type 1 diabetes (T1D) cases [2], latent autoimmune diabetes of adults (LADA) bears close clinical similarity with type 2 diabetes [3, 4]. Thus, T2D and LADA patients receive an initial treatment with oral hypoglycaemic agents (OHA), which leads to variable results ranging from the attenuation to the progression of the phenotype [4].

The onset of severe diabetes in LADA and T2DM is associated with increased impairment of pancreatic islet hormone secretion, which directly impacts body’s glucose homeostasis [5]. The latter is controlled by a concert of two islet antagonising hormones, insulin (secreted by β-cells) and glucagon (α-cells), that ensure glucose clearance from or recruitment into the systemic circulation, respectively. Loss of β-cells, typical to early stages of LADA [6] or later stages of T2DM, is believed to intensify the work of the surviving β-cell population [3], which enhances the expression of autoantibodies by β-cells, in LADA [7]. The depletion of β-cells has been also reported to induce transdifferentiation of other cell types into β-cells [8–10]. An unidentified signal triggering the compensatory mechanism [8] may involve changes in expression of transcription factors such as the increase in *Pdx1* [11], *Pax4* [12], *Ngn3*, *MafA* [13] or loss of *Arx* [14], *Men1* [15], *Dnmt1* [16]. The plasticity of highly committed pancreatic cells, especially the second-largest population of α-cells, is viewed as a tool for regeneration of the β-cell mass [8, 17], an expectation strengthened by reports of therapeutically induced α-cell/β-cell transdifferentiation [15, 18].

An OHA of the thiazolidinedione family with a proven effect on T2DM and LADA progression, rosiglitazone inhibits the activity of PPARγ, increasing insulin sensitivity [19] and glucose uptake by adipose tissue and liver [20]. Thiazolidinediones have been also shown to impact various aspects of β-cell biology [21], such as mitochondrial metabolism [22]. Sulfonylurea tolbutamide targets pancreatic β-cells directly, by inhibiting the intracellular ‘metabolic sensor’ [23], ATP-sensitive K^+^ channels, thereby triggering insulin secretion [24]. Historically the oldest OHA, biguanides are believed to impose their glucose-lowering effect by activating AMP-activated protein kinase (AMPK), which inhibits hepatic glucose production [25], possibly affecting the β-cell function [26].

In the current study, we examined the impact of three oral anti-diabetic agents used for early stage treatment of both T2DM and LADA, rosiglitazone, tolbutamide and metformin, on proliferation and plasticity of pancreatic islet α-cell pool, under the conditions of severe β-cell loss. The latter was modelled in mice bearing an inducible fluorescent label in α-cells (Glu^CreERT2^; ROSA26e-YFP) that were repeatedly treated with low doses of streptozotocin (STZ) to induce apoptosis in β-cells, which is expected to provide a critical signal to compensate for the β-cell loss.

## Materials and methods

### Animals

All experiments, carried out under the UK Animals (Scientific Procedures) Act 1986 and EU Directive 2010/63EU, were approved by the University of Ulster Animal Welfare and Ethical Review Body. Animals were maintained in environmentally controlled rooms at 22 ± 2 °C with a 12 h dark and light cycle and given ad libitum access to standard rodent diet (10% fat, 30% protein and 60% carbohydrate; Trouw Nutrition, Northwich, UK) and water.

#### Glu^CreERT2^;ROSA26-eYFP mice

**Fig. 1:**
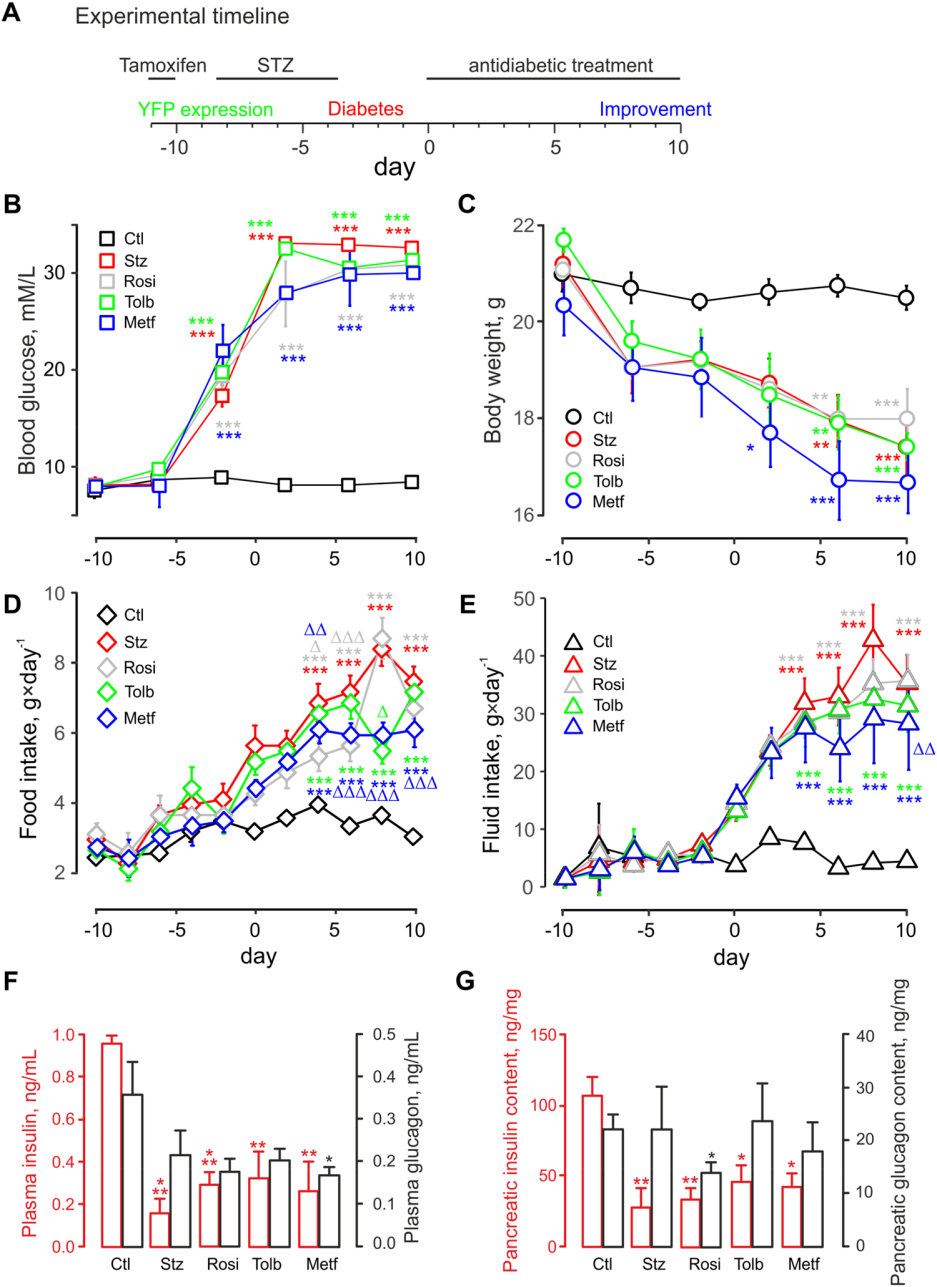
Rosiglitazone, tolbutamide and metformin partially rescue the diabetic phenotype of the streptozotocin-treated mice. *A:* Experimental timeline. Antidiabetic treatment starts on day 0. Tamoxifen is fed to the animals 11 days prior to that to induce the tissue-specific expression of YFP in α-cells. STZ is administered to model type 1 diabetes for 5 successive days, 4 days before the start of the treatment. The ability of the latter to improve the diabetic phenotype is then assayed. *B, C, D, E:* Non-fasting blood glucose (*B*), body weight (*C*), food (*D*) and fluid (*E*) intake of Glu^CreERT2^;ROSA26-eYFP mice, following STZ treatment and the administration of anti-diabetic drugs, as indicated, for groups of *n* = 6 mice each. ‘STZ’, streptozotocin; ‘Rosi’, rosiglitazone; ‘Tolb’, tolbutamide; ‘Metf’, metformin; ‘Ctl’, saline control. *F:* plasma insulin (red) and glucagon (black) *G:* pancreatic insulin (*red*) and glucagon (*black*) content. *F, G* measurements were done on day 10, in separate groups of mice. *p < 0.05, **p < 0.01 and ***p < 0.001 compared to saline control group. Δ*p* < 0.05, ΔΔ*p* < 0.01, ΔΔΔp < 0.001 compared to the STZ group

Nine-week-old male Glu^CreERT2^; ROSA26-eYFP transgenic mice were used to perform all studies. An original colony, developed on the C57Bl/6 background at the University of Cambridge [27], was subsequently transferred to the animal facility at Ulster University and genotyped to assess Cre-ERT2 and ROSA26eYFP gene expression (Table S1). Three days prior to STZ dosing, mice were injected with tamoxifen (i.p. 7 mg/mouse) to activate the tissue-specific expression of yellow fluorescent protein (YFP) in pancreatic islet α-cells (Fig. 1A).

#### Diabetes model and anti-diabetic medications

Our study was designed to evaluate direct effects of rosiglitazone, tolbutamide and metformin on islet morphology and cell transdifferentiation on background of sustained hyperglycaemia. To exclude the effects mediated by changes of insulin sensitivity or blood glucose (that affect islet composition and function [28, 29]), we used mice with insulin-deficient diabetes [30] that was induced by a 5-day course of injections with STZ (Sigma-Aldrich, Dorset, UK; 50 mg/kg body weight daily, i.p.) ( \* MERGEFORMAT Fig. 1A), dissolved in 0.1 M sodium citrate buffer (pH 4.5). The animals that underwent STZ injections and developed hyperglycaemia (non-fasting blood glucose > 10 mM [31]) were then divided into 4 groups (*n* = 6) and treated orally, once a day, with saline vehicle, rosiglitazone (TCI, Oxford, UK; 10 mg/kg), metformin (TCI, Oxford, UK; 100 mg/kg) or tolbutamide (Sigma-Aldrich, Poole, UK; 20 mg/kg) for 10 successive days (Fig. 1A). The doses were selected on the basis of ameliorating milder genetic or high-fat-induced [32] but not STZ-induced [33] forms of diabetes, to elucidate the direct effects on islet cell plasticity. Food and fluid intake were assessed every 2 days, whereas blood glucose and body weight were assessed every 4 days. Non-fasting plasma insulin and glucagon were determined at the termination of the study (day 10).

#### Blood glucose and hormone measurements

Blood samples were collected from the tail vein of animals into ice-chilled heparin-coated microcentrifuge tubes. Blood glucose was measured using a portable Ascencia meter (Bayer Healthcare, Newbury, Berkshire, UK). For plasma insulin and glucagon, blood was collected in chilled fluoride/heparin-coated tubes (Sarstedt, Numbrecht, Germany) and centrifuged using a Beckman microcentrifuge (Beckman Instruments, Galway, Ireland) for 10 min at 12,000 rpm. Plasma was then stored at  – 20 °C. For hormone determination from tissues, samples underwent acid–ethanol extraction (HCl: 1.5% v/v, ethanol: 75% v/v, H_2_O:23.5% v/v). Insulin concentrations were subsequently assessed by an in-house radioimmunoassay [34]. Plasma glucagon and pancreatic glucagon content were measured using glucagon ELISA (EZGLU-30 K, Merck Millipore), or RIA kit (250-tubes GL-32 K, Millipore, USA), respectively.

### Immunohistochemistry and imaging

**Fig. 2:**
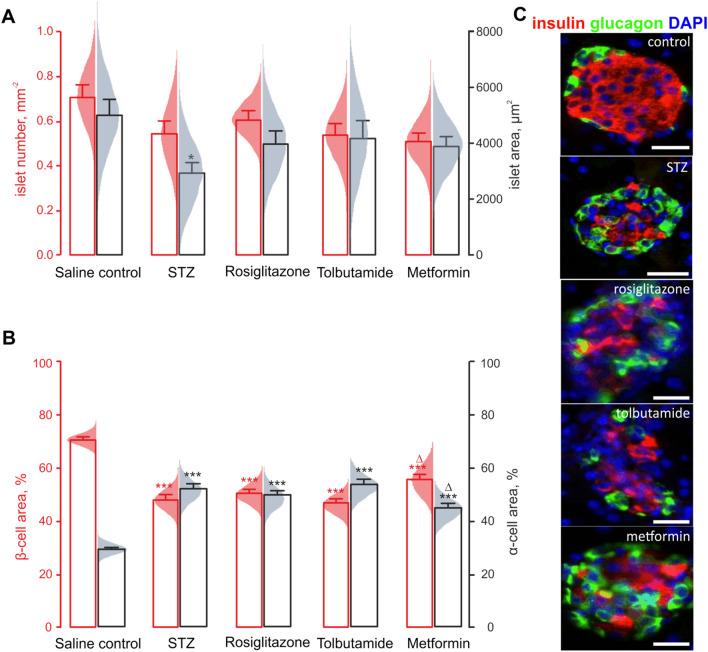
Diabetic phenotype is associated with changes in the islet composition. Impact of the administration of STZ to Glu^CreERT2^;ROSA26-eYFP.mice and subsequent treatment with anti-diabetic drugs, as indicated, on: islet number (black, n = 150 islets from 6 mice) and islet area (red, *n* = 150 islets from 6 mice) (*A*); β- (red, *n* = 150 islets from 6 mice) and α-cell (black, *n* = 150 islets from 6 mice) percentage among the islet cells (*B)*. *C:* Representative immunostaining of mouse pancreatic sections for DAPI (blue), glucagon (green) and insulin (red). *p < 0.05 and ***p < 0.001 compared to the saline control group. Δ*p* < 0.05compared to streptozotocin-treated group. Scale bars: 50 µm

**Fig. 3:**
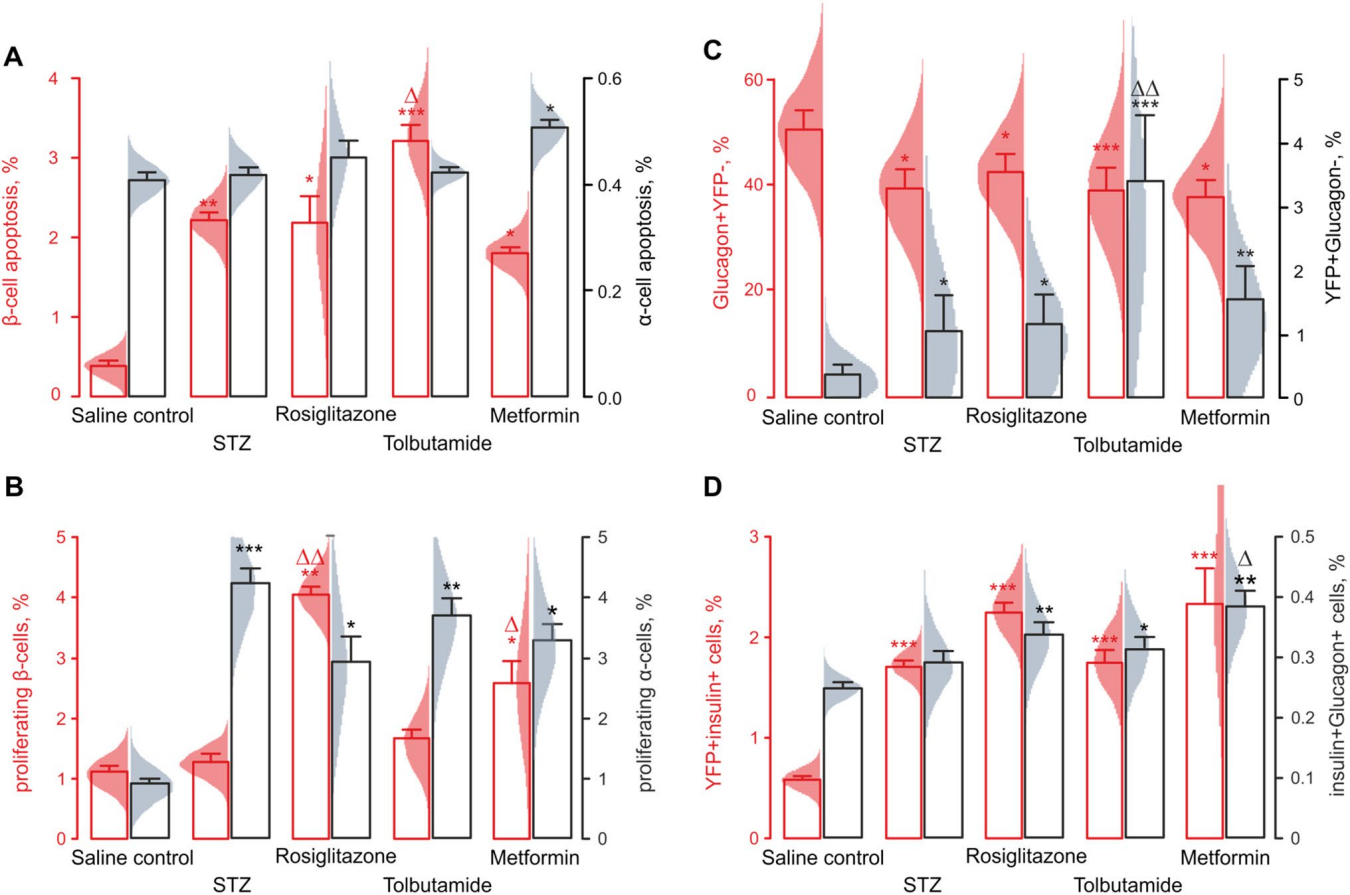
*A, B:* Percentage of β-cells (red, *n* = 60 islets from 6 mice) and α-cells (black, *n* = 60 islets from 6 mice) undergoing apoptosis (*A*), as determined by TUNEL staining, or proliferation (*Β)*, ki67 staining*,* in response to the administration of STZ to Glu^CreERT2^; ROSA26-eYFP.mice and subsequent treatment with anti-diabetic drugs, as indicated. Grey bars in *B* represent the net difference in the proliferating fractions of α- and β-cells. *C, D*: Trans-differentiation of YFP + cells within Glu^CreERT2^;ROSA26-eYFP.mice. The YFP expression, originally specifically induced in α-cells, was detectable within α-cells (*C*, red, *n* = 60 islets from 6 mice), non-α-cells (*C*, black, *n* = 60 islets from 6 mice) and β-cells (*D*, red, *n* = 60 islets from 6 mice) after to the administration of STZ and subsequent anti-diabetic treatment. In addition, double-positive (insulin + glucagon +) cells were detectable (*D*, black, *n* = 60 islets from 6 mice). *p < 0.05, **p < 0.01 and ***p < 0.001 compared to saline control group. Δ*p* < 0.05 and ΔΔ*p* < 0.01, ΔΔΔ*p* < 0.001 compared to STZ-treated group [35]

Following the removal of pancreatic tissue, samples were cut longitudinally and fixed with 4% PFA for 48 h at 4 °C. Fixed tissues were embedded and processed for antibody staining as described [30]. Tissue Sects. (7 μm) were blocked with 2% BSA and incubated with respective primary antibodies overnight at 4 °C, and, subsequently, with appropriate secondary antibodies (Table S2). To stain nuclei, a final incubation was carried out at 37 °C with 300 nM DAPI (Sigma-Aldrich, D9542). To assess cell proliferation and/or apoptosis, co-staining of mouse anti-insulin (Abcam, Cambridge, UK; 1:1000; ab6995) or guinea pig anti-glucagon (PCA2/4, 1:200; raised in-house) with rabbit anti-Ki-67 (1:200; Abcam ab15580) or TUNEL reaction mixture (Roche Diagnostics Ltd, UK) was used. YFP, indicating the α-cell lineage, was detected by with a rabbit anti-GFP antibody (1:1000; Abcam, ab6556) (Table S2), which is reactive against all variants of *Aequorea Victoria* GFP, including YFP. The slides were imaged on an Olympus BX51 microscope, equipped with a 40x/1.3 objective. We aimed to include all the islets visible on the slide in the morphometry analysis, independently of their localisation in relation to other pancreatic structures, with at least 50 cells analysed within each islet cross-section in the per-cell studies (Figs. 2B, 3). The multichannel fluorescence was recorded using DAPI (excitation 350 nm/emission 440 nm), FITC (488/515) and TRITC (594/610) filters and a DP70 camera controlled by Cell^F^ software (Olympus, UK). Images were analysed using ImageJ software. All counts were determined in a blinded manner with 60–150 islets analysed per treatment group, as indicated in the figure legends. The non-stained cells visible in the middle of the islet were not excluded from the computation of the islet area.

### Data analysis and statistics

Statistical analysis was performed using PRISM 5.0 (GraphPad, USA) or R. Values are expressed as mean ± SEM. Comparative analyses between experimental groups were carried out using independent-samples Student's *t* test or (for > 2 samples) a one-way ANOVA with Bonferroni’s post hoc. The difference between groups was considered significant for *p* < 0.05.

## Results

### STZ-induced intake of food and fluid is partially rescued by the anti-diabetic drugs

The treatment with STZ resulted in a progressive diabetic phenotype in the mice, which was reflected by the elevation of blood glucose concentration (Fig. 1B). Non-fasting blood glucose increased in the STZ-treated mice from 8.2 ± 0.4 mM (end of the STZ treatment) to 32.6 ± 0.4 mM 14 days afterwards (7.6 ± 0.7 and 8.4 ± 0.6 mM, respectively, in the control group).

As designed, 10-day administration of rosiglitazone, tolbutamide or metformin had no significant impact on glycaemia (30.8 ± 0.7, 31.3 ± 0.4, 30.0 ± 0.1 mM, respectively) (Fig. 1B). The onset of hyperglycaemia coincided with the 9% decrease in the body weight from 19.2 ± 0.4 g at the end of the STZ treatment (20.7 ± 0.3 g in the control group, n.s.) to 17.4 ± 0.4 g after 14 days (20.5 ± 0.3 g in the control group, *p* < 0.05) (Fig. 1C). 10-day administration of rosiglitazone and tolbutamide had no statistically significant impact on body weight (17.9 ± 0.6, 17.4 ± 0.3 g, respectively, *p* < 0.05 vs control), whereas metformin tended to exacerbate (16.7 ± 0.6 g, *p* < 0.05 vs control) the weight loss (Fig. 1C).

The effects of STZ treatment on the intake of food or fluid by the experimental animals were palpable 4 days post its cessation (day 0, Fig. 1D,E) and were progressively elevating from that point. Both food and fluid intake were significantly attenuated after 4 days of treatment with metformin (Fig. 1D,E), coincident with the decrease in the body weight (Fig. 1C). Neither of the remaining two OHA influenced fluid intake (Fig. 1E); however, rosiglitazone and, at one point, tolbutamide significantly attenuated the intake of food (Fig. 1D).

As a result of the STZ treatment, the non-fasting terminal plasma insulin levels that were measured on day 10 were substantially decreased (0.16 ± 0.06 vs 0.95 ± 0.04 ng/mL in STZ-treated and control groups, *P* < 0.01), whereas the differences between corresponding glucagon levels did not attain statistical significance (0.19 ± 0.07 vs 0.32 ± 0.11 ng/mL) (Fig. 1F). Whilst none of the OHA elevated insulin levels (Fig. 1F), metformin induced a significant decrease of plasma glucagon levels, on the STZ-treatment background (0.15 ± 0.03 vs 0.32 ± 0.11 ng/mL in the control group, *p* < 0.05) (Fig. 1F).

In line with the effect on plasma hormone levels (Fig. 1F), STZ substantially decreased pancreatic content of insulin (27.5 ± 9.9 vs 109.2 ± 8.0 ng/mg of tissue in control, *p* < 0.05), without any appreciable effect on the glucagon content (Fig. 1G). Following subsequent rosiglitazone treatment, the glucagon content was substantially decreased (13.3 ± 3.8 vs 22.7 ± 4.2 ng/mg of tissue in control, *p* < 0.05), whereas tolbutamide or metformin had no effect on this parameter (Fig. 1G).

### The alleviation of the diabetic phenotype is associated with a mild effect on the islet composition

We did not detect any significant alteration in the islet number, in response to any treatments (red in Fig. 2A). At the same time, the observed decrease in plasma and pancreatic insulin (Fig. 1F,G) coincided with the decrease in the average cross-section area of islets in the STZ-treated mice (black in T Fig. 2A). The OHA therapy that followed the STZ treatment resulted in a mild increase in this metric (black in Fig. 2A).

The STZ treatment produced a significant reduction in the relative β-cell area (red/insulin + in Fig. 2B,C) and, respectively, an increase in the relative α-cell area (black/glucagon + in Fig. 2B,C). Remarkably, a 10-day oral administration of metformin, but not rosiglitazone or tolbutamide, counter-acted the effects of the STZ treatment, resulting in small but significant differences in the percentage of β-cells (55 ± 2% vs 48 ± 2% in STZ mice, *p* < 0.05) and α-cells (44 ± 2% vs 51 ± 2% in STZ mice, *p* < 0.05) (Fig. 2B,C). Interestingly, the islets from the STZ-treated animals contained a palpable fraction of cells that did not express insulin or glucagon (Fig. 2C): we need to stress that, among other types, islets contain significant numbers of vascular endothelial cells [35, 36], which are likely to contribute to this phenomenon.

### Oral hypoglycaemic agents increase proliferation but have no effect on apoptosis of β-cells

In line with the report of STZ inducing β-cell apoptosis, when used in small repeated doses [37], we observed a sixfold (2.2 ± 0.1 vs 0.4 ± 0.1% in control mice, p < 0.05) increase in the percentage of β-cells expressing an apoptosis marker, TUNEL, in STZ-treated mice (red, Fig. 3A, Figure S1A). The metformin therapy tended to attenuate the β-cell apoptosis, whereas tolbutamide further increased the expression of TUNEL by β-cells (3.2 ± 0.3% vs 2.2 ± 0.1 in the STZ-treated mice, *p* < 0.05) (red, Fig. 3A). Although the STZ treatment per se has not affected the apoptosis of α-cells (black, Fig. 3A), metformin administered to the STZ-treated animals mildly increased this characteristic (0.5 ± 0.1% vs 0.4 ± 0.1% in the STZ-treated group, *p* < 0.05) (black, Fig. 3A, Figure S1A).

The pro-apoptotic effect of the STZ treatment did not affect the percentage of proliferating β-cells, as was assayed via Ki-67 staining (red, Fig. 3B, Figure S1B). This metric was increased by subsequent treatment with rosiglitazone or metformin (4.0 ± 0.2% and 2.5 ± 0.4% respectively, vs 1.3 ± 0.1% in the STZ-treated group, *p* < 0.05). The STZ treatment produced a fivefold increase in the fraction of proliferating α-cells (black in Fig. 3B, Figure S1B), which was not affected by any of the OHA (black in Fig. 3B).

### Long-term administration of oral hypoglycaemic agents does not affect α-/β-cell transdifferentiation

The key feature of the animal model used in this study, the Glu^CreERT2^; ROSA26-eYFP mice, is the tissue-specific targeting (pancreatic α-cells) and the inducible nature of the expression of YFP. When co-detected with anti-glucagon antibodies, 20 days post-induction of the targeted YFP expression, the islets from these mice had only a small fraction of YFP + cells that did not express glucagon (0.3 ± 0.1%) (black, Fig. 3C, Figure S1C). The YFP + cell percentage was increased almost threefold after the STZ treatment (0.8 ± 0.4%, *p* < 0.05 vs control) and further potentiated by tolbutamide (2.7 ± 0.8%, *p* < 0.005 vs control, *p* < 0.01 vs STZ) but not rosiglitazone or metformin (0.9 ± 0.4%, *p* < 0.05 vs control and 1.2 ± 0.4%, *p* < 0.01 vs control, respectively) (black, Fig. 3C, Figure S1C). Of note, we were unable to detect YFP in almost half of glucagon^+^ cells, which we believe to reflect a technical feature of the anti-GFP antibody staining (red in Fig. 3C).

The percentage of the YFP^+^insulin^+^ cells was low in the experimental animals with α-cell-specific targeting of YFP (0.6 ± 0.1% in the control group). The treatment with STZ however triplicated this number (1.7 ± 0.1%) (red, Fig. 3D, Figure S1D). Neither of the OHA was able to further enhance the commitment of the YFP + cells towards the insulin lineage (red, Fig. 3D). At the same time, the administration of each of the OHA, following the STZ treatment, increased the percentage of bi-hormonal (insulin + glucagon +) cells (black, Fig. 3D, Figure S1D). The size of this small cell subpopulation was unaffected by the STZ treatment (0.27 ± 0.01% vs 0.25 ± 0.02% in the control group), whereas subsequent rosiglitazone (0.33 ± 0.02%), tolbutamide (0.31 ± 0.02%) and metformin (0.38 ± 0.03%) administration substantially expanded it (black, Fig. 3D).

## Discussion

We probed the mechanisms whereby the oral hypoglycaemic agents may partially compensate for the selective apoptotic damage of β-cells. In our hands, pancreatic β-cell population was partially replenished via increased proliferation, in response to metformin or rosiglitazone, whereas tolbutamide exacerbated apoptosis, arguably by putting an extra demand on insulin production and secretion mediated by cytosolic Ca^2+^ [38].

### Diabetic phenotype of the mice

The diabetes model and the OHA dosage were designed to resolve the *direct* effects of the OHA on pancreatic islet cell plasticity [39]. We have opted for repeated injections of small doses of STZ [9, 40] over high-fat diet or leptin receptor deficiency animal models of diabetes to enable cell lineage tracing and rule out any indirect effects, mediated by changes in insulin sensitivity or blood glucose, that may impact the islet plasticity. The model animals displayed stably elevated glycaemia and reduced body weight (Fig. 1B,C), whereas the three treatments, at the doses chosen, affected only food and fluid intake (Fig. 1D,E). Notably, the doses used compare well with daily human recommended doses, given the differences in the pharmacokinetics of the three drugs in the mouse and human systems [41–43].

### Islet mass, morphology, apoptosis and proliferation of β- and α-cells

A side effect of the OHA therapy, lowering of the systemic glucagon in response to metformin (Fig. 1F), is unlikely to reflect the depletion of the α-cell population due to its transdifferentiation or apoptosis, as α-cells are well in excess, in rodent islets [35, 44]. The phenomenon could have stemmed from the elevation of circulating GLP-1 levels, reported to be induced by metformin [45]. Another possible explanation for this effect is the activation of the intra-islet GLP-1 secretion system [46–48], under the conditions of the STZ treatment [9, 49]. The likely mechanism for that is the acquisition of the proconvertase PC1/3 activity by α-cells [46], with a subsequent shift in the α-cell secretory output from glucagon to GLP-1. In line with the reported cytostatic effect of metformin [50] that, in our hands, stimulated apoptosis in α-cells (but, surprisingly, given earlier reports [51], not in β-cells, Fig. 3A), the elevation of intra-islet and circulating GLP-1 could explain partial recovery of the ratio of β- and α-cells (Fig. 2B), presumably by upregulating β-cell proliferation [9, 30]. Notably, rosiglitazone, reported to increase the β-cell mass by reversing the apoptosis [52], was not efficient in doing so in our model (Fig. 3A). In our hands, it induced a fourfold increase of the proliferating β-cells, in line with the previous reports [52].

### Effects of OHA on alpha cell transdifferentiation

The STZ-induced β-cell injury per se resulted in a detectable expression of insulin and a loss of expression of glucagon by YFP^+^ cells (Fig. 3C), reflecting the α-cell population before the STZ treatment. The fact that none of the OHA affected the co-expression of insulin and YFP on a per-cell basis suggests the lack of a role in regulating α-/β-cell transdifferentiation (Fig. 3D).

In the present study, a small but detectable number of bi-hormonal [8] cells was evident after the STZ treatment, followed by administration of metformin (Fig. 3D). This effect can be explained by non-pancreatic signals [53] that may induce α-cell transdifferentiation. On the contrary, the de-differentiation of α-cells by tolbutamide (Fig. 3C), likely to result from a direct effect on the K_ATP_ channels, was not associated with any β-cell phenotype.

### Relative merits of different OHAs

Since sulphonylureas are actively prescribed for diabetes, further elucidation of their global effects on islet function is highly relevant. No previous study has reported, to our knowledge, on the effects of this drug class on islet cell transdifferentiation. Our data with tolbutamide are important in revealing that not only does the sulphonylurea lack beneficial effects on islet plasticity (unlike the two other classes of OHA) but that it exerts adverse effects on β-cell health and apoptosis. This can be viewed as a significant limitation of first-line sulphonylurea therapy and would suggest that incretin mimetics which have recently shown to have positive effects on β-cell transdifferentiation, apoptosis and proliferation [9, 29] would be a better therapeutic option for direct β-cell actions.

## Conclusion

Alongside peptide hormones [9, 40], small molecules have been shown to induce transdifferentiation of pancreatic α-cells into β-cells. Metformin and rosiglitazone but not tolbutamide promoted the restoration of the β-cell pool via proliferation, with none of the three oral anti-diabetic drugs affecting the α-cell transdifferentiation induced by the loss of β-cells. In contrary, metformin decreased the islet α-cell population via apoptosis, whereas tolbutamide, in turn, enhanced apoptosis in β-cells. Whether these drugs impose similar effects in humans, alongside the reported antioxidant [54] and insulinotropic [55] activity, remains the matter of future research.

## Supplementary Information

Below is the link to the electronic supplementary material.

Supplementary file1 (DOCX 1758 KB)

## Abbreviations

T1D (T2D): Type 1 (2) diabetes mellitus
LADA: Latent autoimmune diabetes of adults
YFP: Yellow fluorescent protein
OHA: Oral hypoglycaemic agent
STZ: Streptozotocin
PPARγ: Peroxisome proliferator-activated receptor type γ
K_ATP_ channel: ATP-sensitive potassium channel
AMPK: AMP-activated protein kinase
