# Supplementary figures and images for: Effects of first-line diabetes therapy with biguanides, sulphonylurea and thiazolidinediones on the differentiation, proliferation and apoptosis of islet cell populations
Source: J Endocrinol Invest. 2021 Jun 30;45(1):95–103. doi: 10.1007/s40618-021-01620-6 (PMC8741670; doi:10.1007/s40618-021-01620-6)

**
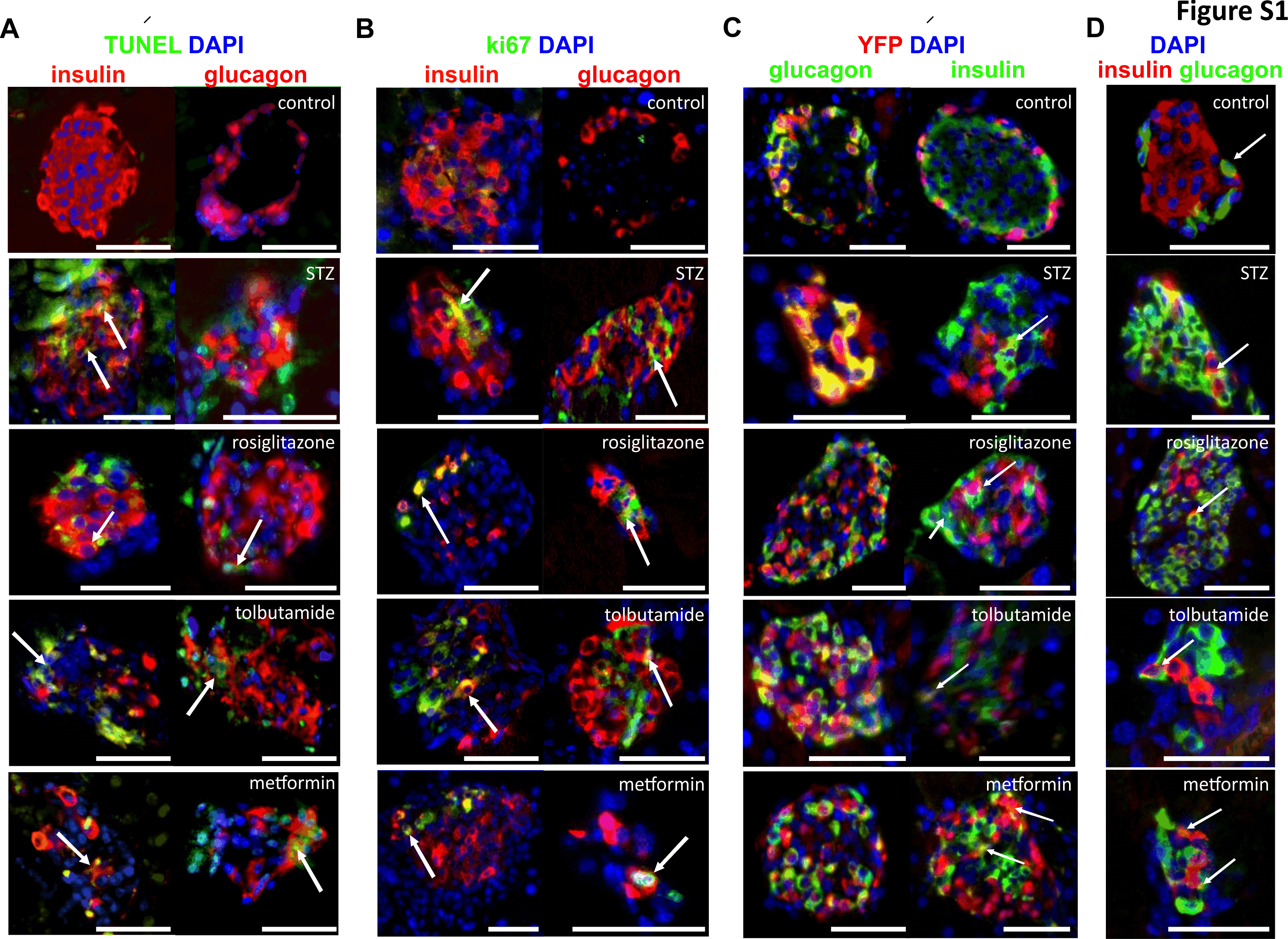
**

Supplement: Supplementary file 1 — Supplementary file1 (DOCX 1758 KB) [file 40618_2021_1620_MOESM1_ESM.docx]
